# Incidence of microcytosis in hyperthyroid cats referred for radioiodine treatment

**DOI:** 10.1177/1098612X20983973

**Published:** 2021-01-19

**Authors:** Claudia Gil-Morales, Marta Costa, Kathleen Tennant, Angie Hibbert

**Affiliations:** 1 School of Veterinary Sciences, University of Bristol, Bristol, UK; 2 Dick White Referrals, Station Farm, Newmarket, UK; 3 The Feline Centre, Langford Vets, University of Bristol, Bristol, UK

**Keywords:** Hyperthyroidism, microcytosis, iron, radioiodine

## Abstract

**Objectives:**

The study aimed to document the incidence of erythrocyte microcytosis in a population of hyperthyroid cats referred for radioiodine (RAI) treatment. Microcytosis has been observed but not described in feline hyperthyroid patients and is associated with hyperthyroidism in humans.

**Methods:**

Retrospective clinicopathological data were collected for cats undergoing RAI between January and December 2017. Microcytosis was defined as mean cell volume (MCV) <41.3 fl using the ADVIA 2120 haematology analyser (Siemens) and identified on blood smear examination by a haematology laboratory scientist or board-certified specialist in veterinary clinical pathology. Hyperthyroidism was classified as mild (total thyroxine [TT4] 60–124.9 nmol/l), moderate (TT4 125–250 nmol/l) or severe (TT4 ⩾251 nmol/l) immediately before RAI. Data were analysed descriptively and using a Pearson correlation coefficient to test the relationship between TT4 and microcytosis, and time elapsed between first diagnosis and MCV.

**Results:**

There were 41 female and 37 male cats with an age range of 7.2–20.8 years. Most cats were non-pedigree (98.7%). Microcytosis (median MCV 39.8 fl, interquartile range 32.3–41.2) was present in 29.5% (23/78) of the cats. Of the 23 microcytic samples, 86.9% (20/23) were confirmed as such on smear examination. Of mildly, moderately and severely hyperthyroid cats, 23% (6/26), 28.1% (9/32) and 40% (8/20) were microcytic, respectively. Two microcytic cats had low red blood cell counts (<6 × 10^12^/l) and low haemoglobin concentration (<8.2 g/dl). There was no correlation between TT4 or time elapsed from first diagnosis and MCV. Microcytosis resolved in 77.7% (7/9) of cases with follow-up. One microcytic cat had significant comorbidities (portosystemic shunt).

**Conclusions and relevance:**

Microcytosis was present in a significant proportion of hyperthyroid cats, most without clinically significant comorbidities, and resolved in some following RAI.

## Introduction

Hyperthyroidism is the most common endocrine disease of domestic cats, affecting especially middle-aged and senior cats.^1,2^ Its classic clinical features were well described decades ago after hyperthyroidism was first reported in 1979,^3^ and since then ongoing research has mainly focused on its diagnosis and treatment. The population of cats diagnosed with this disease may have changed owing to the increased awareness of hyperthyroidism by clinicians and earlier diagnosis, facilitated by the inclusion of total thyroxine (TT4) in routine senior screening panels. In a recent study, 24.3% of the cats referred for radioiodine (RAI) treatment were diagnosed incidentally.^4^

Microcytosis, defined as the presence of small red blood cells compared with a reference population, can be observed with several diseases including iron deficiency, iron-restricted haematopoiesis (such as in hepatic disease, chronic kidney disease [CKD] or chronic inflammatory disease) and vitamin B_6_ deficiency. It may be breed-associated (Abyssinian) or a spurious result (eg, hyponatraemia, excessive EDTA).^5–7^ In human medicine, microcytosis has been associated with hyperthyroidism, with a variable incidence from 42% to 87.7% in different studies.^8,9^ Erythrocytosis and microcytic anaemia are also common in human hyperthyroidism, although macrocytosis has also been reported.^9^ Thyroid hormone receptors have been found in human haematopoietic cells and their expression depended on the patient thyroid hormone status, suggesting a role of these hormones in haematopoiesis.^8^

In early reports of feline hyperthyroidism, macrocytosis was reported to be common,^1,2^ although this was considered rare in similar studies.^10^ Microcytosis has been clinically observed in feline patients referred to our centre for assessment of suitability for RAI treatment.

The objective of this study was to describe the incidence of microcytosis in a population of hyperthyroid cats referred for RAI treatment. Secondary aims were to describe concurrent haematological abnormalities, comorbidities that could cause iron-restricted haematopoeisis, whether resolution of microcytosis occurred following RAI treatment and finally to determine whether there was a correlation between microcytosis and TT4 or time elapsed from first diagnosis.

## Material and methods

### Study population

Medical records of cats that received RAI treatment at the Feline Centre, Langford Vets (Bristol, UK), between January and December 2017 were reviewed. Hyperthyroidism was diagnosed prior to referral based on increased TT4 or free thyroxine (fT4) above the reference interval for the laboratory used. Cats were included if there was a complete record of history, diagnostic tests performed on RAI assessment, with haematology and TT4 levels measured immediately prior to and after RAI treatment. Ethical approval was granted by the Animal Welfare and Ethics Review Body at the University of Bristol (VIN/18/024).

### Procedures

Retrospective data were collected regarding signalment, previous medical history, physical examination findings, TT4 or TT4 and fT4 levels at diagnosis, TT4 levels and haematology results immediately prior to RAI treatment (when antithyroid medication or iodine-restricted diet had been withdrawn) and TT4 levels and haematology results post-treatment (before discharge from the hospital at 2–4 weeks post-RAI, and any follow-up tests available post-discharge). Time elapsed from first diagnosis was defined as the time interval between the initial diagnosis of hyperthyroidism and RAI treatment. Additional data collected during RAI suitability assessment (performed 4–6 weeks before RAI treatment) for all cats were serum biochemistry, urinalysis, systolic blood pressure, retinal examination, abdominal ultrasonography, thoracic radiography and echocardiogram findings. In some cases, at the clinician’s discretion, further investigations were performed including serum cobalamin, folate, trypsin-like immunoreactivity and pancreatic lipase immunoreactivity measurement, bile acid stimulation test (BAST), fine-needle aspiration, thoracic CT and/or scintigraphy.

Microcytosis was defined as mean cell volume (MCV) <41.3 femtolitre (fl) using the ADVIA 2120 haematology analyser (Siemens) and as identified on blood smear examination performed by a haematology laboratory scientist (n = 3) or board-certified specialist in veterinary clinical pathology (n = 1). The MCV reference interval used was established by Siemens for the ADVIA 2120 haematology analyser following analysis of 100 feline samples. The degree of microcytosis was evaluated on blood smear and classified as mild, moderate or severe according to criteria described previously.^11^ Other red blood cell abnormalities noted, such as anaemia, erythrocytosis or acanthocytosis, were recorded.

Cats were classified according to TT4 levels immediately prior to RAI treatment as mildly (TT4 60.1−124.9 nmol/l), moderately (TT4 125−250 nmol/l) or severely (TT4 ⩾251 nmol/l) hyperthyroid.^10^

The presence of comorbidities (based on previous history and assessment findings) was recorded and classified as gastrointestinal, hepatic, renal, respiratory, cardiac and other disease. CKD was defined according to International Renal Interest Society (IRIS) guidelines as fasting serum creatinine ⩾140 µmol/l, urine specific gravity <1.035 and/or structural ultrasonographic renal changes.

### Statistical analysis

A computerised statistical software package (SPSS Version 24; IBM) was used for statistical analysis. Data were analysed descriptively and presented as median (interquartile range [IQR]; 25th−75th percentile or range) where appropriate. The significance level was set at *P* <0.05. Pearson correlation coefficient was used to test the relationship between TT4 and MCV, and time elapsed from first diagnosis and MCV in the whole study population and in the microcytic population.

## Results

There were 41 spayed female cats and 37 castrated male cats and the age range was 7.2−20.8 years (median 13.2 years; IQR 11.02−15 years). Most cats were non-pedigree (98.7%), with only one pedigree breed cat (Norwegian Forest Cat).

Immediately prior to RAI treatment, 26 cats had mild hyperthyroidism (TT4 median 84.9 nmol/l, IQR 65.5−104.5 nmol/l), 32 cats had moderate hyperthyroidism (TT4 median 177 nmol/l, IQR 153.5−224.3 nmol/l) and 20 cats had severe hyperthyroidism (TT4 median 328 nmol/l, IQR 262.5−409.8 nmol/l). One cat with mild hyperthyroidism had TT4 within normal limits at the time of treatment, so the diagnosis of hyperthyroidism was based on an elevated fT4 (TT4 49 nmol/l reference interval (RI) 15–60 nmol/l; fT4 41.8 pmol/l, RI: 9–30 pmol/l; thyroid-stimulating hormone [TSH] not available) and compatible clinical signs.

Microcytosis (median MCV 39.8 fl, range 32.3−41.2 fl, reference interval 41.3−52.6 fl) was present in 29.5% (23/78) of the cats. Microcytosis was present both before and immediately post-treatment in 22/23 cats. Of mildly, moderately and severely hyperthyroid cats, 23% (6/26), 28.1% (9/32) and 40% (8/20) were microcytic, respectively (Table 1). Of the non-microcytic cats, median MCV was 43 fl (range 41.4−48.5 fl).

**Table 1:** Summary of degree of hyperthyroidism and presence of microcytosis immediately prior to and post-radioiodine treatment in a referral population of hyperthyroid cats

Degree of hyperthyroidism	Number of cats	Median TT4 in nmol/l (range)	Percentage of cats microcytic pre-treatment based on haematology	Percentage of cats microcytic pre-treatment based on blood smear	Number of cats with long-term resolution of microcytosis post-treatment when follow-up available
Mild	26	84.9 (22.5–121)	23% (6/26)	23% (6/26)	2/2
Moderate	32	177 (125–250)	28.1% (9/32)	25% (8/32)	2/4
Severe	20	328 (256–1106)	40% (8/20)	30% (6/20)	2/2

TT4 = total thyroxine

Of the 23 cats with microcytosis, 86.9% (20/23) had confirmation of this on blood smear examination. The degree of microcytosis was graded as mild in 65% (13/20) and moderate in 35% (7/20) of the smears examined. There were no samples with severe microcytosis.

Of the 23 cats with microcytosis, 26.1% (6/23) had mild hyperthyroidism (TT4 median 72.5 nmol/l, IQR 54.8−104 nmol/l), 39.1% (9/23) had moderate hyperthyroidism (TT4 median 189 nmol/l, IQR 152−230 nmol/l) and 34.8% (8/23) had severe hyperthyroidism (TT4 median 398 nmol/l, range 259.5−683 nmol/l). A significant correlation between TT4 levels and MCV measured immediately prior to RAI treatment was not identified in the microcytic population (*r* = 0.301, *P* = 0.16) or in the whole study population (*r* = −0.115, *P* = 0.317).

The median time from diagnosis of hyperthyroidism to RAI treatment was 133 days (IQR 112−209 days). There was no significant correlation between the duration of hyperthyroidism and MCV levels in the microcytic cats (*r* = −0.369, *P* = 0.08) or in the whole population (*r* = 0.007, *P* = 0.95).

None of the cats included in the study had erythrocytosis or macrocytosis. Only two microcytic cats had low red blood cell counts (<6 × 10^12^/l) and low haemoglobin concentration (<8.2 g/dl). One of these had mild hyperthyroidism and microcytosis was documented prior to RAI treatment (MCV 40.2 fl). The second cat had moderate hyperthyroidism and microcytosis prior to and immediately after receiving RAI treatment (MCV 39.9 fl). In both cases, anaemia and microcytosis resolved following RAI (33 and 64 days after treatment, respectively). One non-microcytic cat had mild anaemia, which was persistent following RAI treatment. This may have been secondary to other comorbidities present (chronic enteropathy, less likely IRIS stage 1 CKD).

Other red blood cell abnormalities noted on blood smear examination included the presence of acanthocytosis in 21/78 (26.9%) cats. Of these, nine had microcytosis. There was no correlation between the degree of acanthocytosis and TT4 (*r* = 0.132, *P* = 0.57) or time elapsed from first diagnosis (*r* = −0.148, *P* = 0.53).

**Figure 1:**
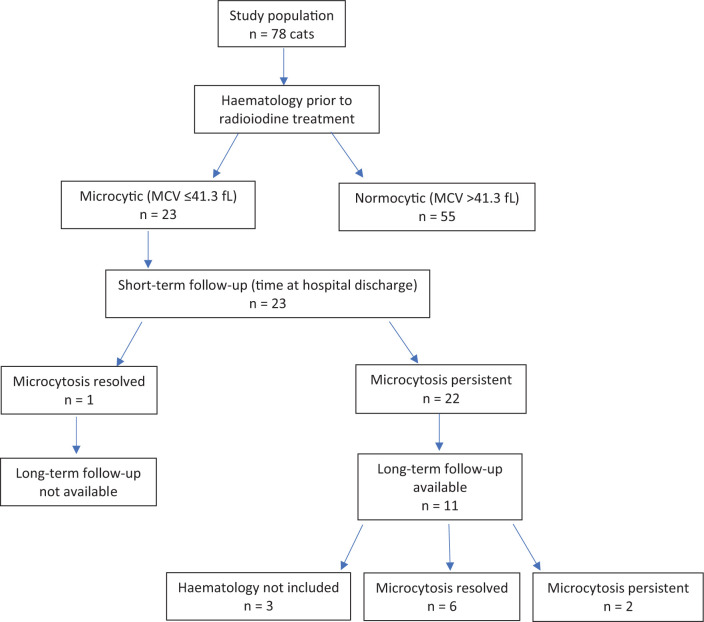
Flowchart demonstrating the short- and long-term outcome of hyperthyroid cats treated with radioiodine in relation to their microcytosis status. MCV = mean cell volume

MCV normalised immediately after RAI treatment in only one of the microcytic cats (haematology sample taken at the time of discharge). Long-term follow-up was available in 11 of the microcytic cats. Of these, full haematology was not performed in three cases post-discharge, leaving a long-term follow-up population of eight cats. Microcytosis resolved in 75% (6/8) of these. The short- and long-term outcome of these cats are summarised in Figure 1. Blood sampling was performed between 54 and 605 days (median 135 days) after receiving RAI treatment. Two cats were persistently microcytic (median MCV 38.1 fl). Blood sampling was performed in these two cases between 46 and 266 days.

Comorbidities were documented in all microcytic cats; however, clinical significance was not apparent at the time of writing for these abnormalities (Table 2). Forty-eight percent of the microcytic cats (11/23) had small intestinal wall thickening, of which two had vomiting as a presenting sign at the time of diagnosis of hyperthyroidism and one had hypocobalaminaemia. Vomiting resolved in these two cats after normalisation of TT4. A high proportion of cats, 91.3% (21/23), had mild chronic kidney disease (IRIS stage 1) based on ultrasonographic renal appearance, serum biochemistry and full urinalysis.

**Table 2:** Prevalence of comorbidities in hyperthyroid cats identified during radioiodine suitability assessment in the whole study population and in microcytic cats

Comorbidities	Number of cats	Number of microcytic cats	Examples (number within whole population, number within microcytic population)
Gastrointestinal	35/78	11/23	Small intestinal wall thickening (35/78,11/23)
Hepatic	60/78	15/23	Hypoechoic nodules (38/78,12/23) Hyperechoic parenchyma (32/78, 5/23) Gallbladder sludge (10/78, 2/23) Biliary cysts (8/78, 0/23) Tortuous common bile duct (2/78, 0/23) Portosystemic shunt (1/78,1/23)
Renal	62/78	21/23	Loss of corticomedullary definition (44/78,14/23) Hyperechoic renal cortex (11/78, 5/23) Cortical infarcts (12/78, 5/23) Medullary rim sign (7/78, 5/23) Pyelectasia (2/78, 2/23) Nephrolith (1/78,1/23)
Respiratory	26/78	7/23	Moderate/marked bronchointerstitial pattern (2/78, 2/23) Mild bronchointerstitial pattern (7/78,1/23) Mild interstitial pattern (6/78, 2/23) Mild bronchial pattern (11/78,1/23) Soft tissue lung mass (1/78, 0/23)
Cardiac	21/76*	15/21*	Left ventricular hypertrophy (37/76,14/21) Mild left atrial enlargement (18/76, 6/21) Mild diastolic dysfunction (27/76, 6/21)
Other	33/78	13/23	Mesenteric lymphadenomegaly (9/78, 6/23) Heterogeneous pancreas (10/78, 4/23) Heterogeneous spleen (9/78, 2/23) Adrenomegaly (7/78, 3/23)

* Full echocardiography was not available in two cases

## Discussion

Red blood cell microcytosis was relatively common in this referral population of hyperthyroid cats, where 29.5% (23/78) had reduced MCV. Most of these cats also had microcytosis in the absence of anaemia (21/23) and evidence of microcytosis on blood smear evaluation, which was more commonly mild (65%). These results are in line with our clinical observations, where microcytosis had been observed prior to administering RAI treatment. All cats had received an iodine-restricted diet or antithyroid medication prior to RAI treatment; however, red blood cell volume or shape changes secondary to these have not been reported to date.^12–14^ When microcytosis was incidentally encountered in a population of hyperthyroid cats, this was present before and after receiving an iodine-restricted diet.^15^

To our knowledge, a tentative association between feline hyperthyroidism and microcytosis has only been reported in one previous study, where the latter was noted incidentally in at least 25% of the cats.^15^ A causal relationship was not established. This contrasts with the available literature regarding clinicopathological findings in feline hyperthyroidism, where macrocytosis has been previously reported in 31–45% of these cats.^1,2^ In these studies, cats with different degrees of hyperthyroidism were included. Feline leukaemia virus (FeLV) status or laboratory sampling information (haematology analyser used, blood smear examination) were not available to exclude other causes of macrocytosis. In a contemporaneous study from a different hyperthyroid cat population, there were minimal changes in the red blood cell parameters and macrocytosis was rare.^10^

In the human literature, the main haematological findings reported in hyperthyroidism are erythrocytosis, anaemia and alterations in red blood cell volume (both macro- and microcytosis).^8^ Microcytic anaemia has been associated with hyperthyroidism more commonly than in other thyroid function states,^16^ more specifically in conjunction with Graves’ disease (formerly known as Graves’ disease anaemia) and iron deficiency anaemia of celiac disease,^17^ both having a possible immune-mediated mechanism. The pathogenesis of microcytosis in cats may be different as feline hyperthyroidism is more commonly caused by thyroid gland adenoma; additionally, comorbid disease is more likely in an older cat population. However, in most human papers, the underlying mechanism behind hyperthyroidism was not specified. The incidence of microcytosis in these studies is very variable (42–87.7%) and it has been observed in the absence of anaemia or reduced transferrin saturation.^18^

In this population, there was a higher incidence of microcytosis in cats with severe hyperthyroidism (40%) in comparison with mild and moderate hyperthyroidism (23% and 28.1%, respectively). However, no significant correlation was found between TT4 and MCV levels. A negative correlation between fT4 and MCV values has been previously described in humans.^9^ There was no relationship either between MCV levels and time elapsed from first diagnosis. The small population size, likely underpowered, may have influenced the lack of identification of statistical association between these variables.

Other haematological findings previously seen in feline hyperthyroid patients, such as erythrocytosis and anaemia, were not common. Only two microcytic cats were mildly anaemic, which resolved following RAI treatment. This has been reported following RAI treatment in most human cases (79%).^19^ Acanthocytosis was identified in 26.9% of all cats, with no concurrent evidence of fragmentation injury on blood smear examination. This finding is of unknown significance, although acanthocytosis can be secondary to changes in the red blood cell membrane, which was initially postulated as a cause for microcytosis in human hyperthyroidism.^20^ Acanthocytosis has been mentioned before in hyperthyroid cats as an infrequent finding,^21^ while it is relatively common in human hypothyroidism.^22,23^ A possible relationship is difficult to establish, although acanthocytosis was persistent in one hyperthyroid cat despite receiving carbimazole treatment.^21^

When follow-up was available post-discharge, most cats had resolution of microcytosis (75%, 6/8). The two cats with persistent microcytosis were analytically hypothyroid and had follow-up blood analysis 46 and 266 days post-treatment. Neither were anaemic, they had moderate hyperthyroidism at diagnosis and only minor findings were identified on their RAI pre-treatment assessment, including mildly prominent mesenteric lymph nodes (n = 2), IRIS stage 1 CKD (n = 2), a heterogeneous pancreas (n = 1) and thickening of the small intestinal muscularis (n = 1). Three of the six cats where microcytosis resolved were euthyroid, while three remained biochemically hypothyroid (54–605 days of follow-up, no TSH measurements available). Resolution of anaemia and/or microcytosis in most cases has been reported in the human literature following treatment with carbimazole, methimazole or RAI.^9,17,19^

Most cats had no clinically significant comorbidities (eg, no associated clinical signs obvious to owners). This would be expected as all patients’ histories were checked prior to referral to exclude patients with comorbidities that might preclude RAI treatment (eg, IRIS stage 3 CKD, mediastinal mass, congestive heart failure) and cats failing the suitability assessment owing to major life-limiting comorbidities were not included within this study. It is possible that some of the comorbidities present were age-related rather than being directly associated with hyperthyroidism. Previous literature reported subclinical comorbidities as common in hyperthyroid cats (minor and major diseases in 40% and 30% of cases, respectively),^24^ with a high prevalence of CKD (32.9%)^4^ and concurrent non-renal disease (18%).^25^ Abnormalities on ultrasound examination are common in hyperthyroid cases (36.1–82% of cases),^25,26^ and the findings in this population are similar to previous studies, where kidney abnormalities, including reduction in corticomedullary definition, small intestinal thickening and presence of liver nodules were relatively common.^26^ One microcytic cat (15-year-old neutered female domestic shorthair) had an incidental portosystemic shunt identified ultrasonographically and suspected underlying enteropathy based on the presence of hypocobalaminaemia, thickened jejunal wall and chronic vomiting. It is possible that the microcytosis in this case was caused by functional iron deficiency secondary to these comorbidities.

Microcytosis has also been described previously as a spurious result. Hyponatraemia can cause a falsely low MCV in analysers such as ADVIA; however, none of the cats were hyponatraemic. Excess EDTA could artefactually have reduced the MCV values, although no EDTA excess changes were observed on blood smear examination. In dogs with portosystemic vascular anomalies, storage of EDTA samples has been reported to mask microcytosis.^27^ This has not been proven in cats, but could be a reason why microcytosis is not commonly noted in first-opinion hyperthyroid populations. The time from blood sampling to laboratory receive time was not standardised in this population; however, in all cats it was below 24 h, with the reference laboratory carrying out the tests on site. Future research into the storage effect of feline EDTA blood samples on MCV measurement could be of interest.

An age-matched control population was not included in the design of this study, and therefore age cannot be excluded as a confounding factor despite microcytosis not being previously described as a common finding in routine health screening in senior cats.^28^ It would be difficult as well to fully exclude comorbidities in a control population of healthy senior cats without extensive screening.

Investigations into causality of microcytosis are beyond the scope of this study; however, it is worth noting that the underlying cause in hyperthyroid human patients is still not known. Possible mechanisms have been postulated, including premature aging of circulating erythrocytes,^16^ increased rate of erythropoiesis by the bone marrow due to thyrotoxicosis,^17^ enteric iron malabsorption^7^ and alteration in the lipid composition of the red blood cell membrane.^20^ Serum iron levels and total iron binding capacity were normal in most of the microcytic hyperthyroid human patients tested (72–100%).^16,29^ Serum iron profiles may be of value in hyperthyroid cats to assess the potential impact of thyroidal disease on iron metabolism. Reticulocyte indices could be measured as well, as these may reflect more accurately the current iron status in cats, as previously seen in dogs.^30^

Limitations of this study include its retrospective nature, which limited the number of cases with follow-up available. This highlights the lack of adherence to the recommended monitoring suggestions post-RAI in this population of cats, which is particularly important to identify diseases that may arise following treatment, such as unmasking of CKD, iatrogenic hypothyroidism and/or systemic hypertension. The population data were also not homogeneous, as although most cases had similar diagnostic tests performed, some investigations that may have ruled out causes of microcytosis were only performed at the clinicians’ discretion if clinically indicated (eg, BAST, serum cobalamin) and serum iron testing was not performed retrospectively. This population only includes cases referred for RAI treatment and may not be representative of the whole population of hyperthyroid cats. Inter- and intra-observer agreement on blood smear examination findings was not evaluated due to the retrospective nature of this study. Although it is possible that this could have influenced our results, there was good consensus between MCV and microcytosis identified on blood smears (86.9% of the samples with reduced MCV were also identified as microcytic on blood smear examination).

Further prospective research in a larger cohort of hyperthyroid cats may be warranted, including standardisation of laboratory variables and screening for comorbidities, reticulocyte indices measurement and funded follow-up blood testing to encourage client participation post-discharge. Measuring serum iron levels could be considered to investigate a possible relationship between microcytosis and hyperthyroidism in cats with and without comorbidities, as the latter could cause microcytosis secondary to functional iron deficiency.

## Conclusions

Microcytosis was present in a significant proportion of hyperthyroid cats referred for RAI, most without clinically significant comorbidities, and resolved in some following RAI treatment. There was no relationship identified between microcytosis and severity or duration of hyperthyroidism in this population. The mechanism of a possible relationship between hyperthyroidism, compromised haematopoiesis and microcytosis is not understood, and research into iron-restricted haematopoiesis in feline hyperthyroidism may be of future interest.
